# Phenotypes of carriers of two mutated alleles in major cancer susceptibility genes

**DOI:** 10.1007/s10549-024-07454-z

**Published:** 2024-08-05

**Authors:** Yael Laitman, Anni Niskakoski, Rinal Bernstein-Molho, Lotta Koskinen, Daniel Rabina, Juha Koskenvuo, Eitan Friedman

**Affiliations:** 1 The Oncogenetics Unit, The Institute of Human Genetics, Sheba Medical Center, Tel-Hashomer, Israel; 2 The Innovation Arm, Assuta Medical Center, Tel Aviv, Israel; 3 The Oncogenetics Service, Assuta Medical Centers, Tel Aviv, Israel; 4 The Center for Genetics and Early Detection, Assuta Medical Centers, Tel Aviv, Israel; 5 The Faculty of Medical and Health Sciences, Tel-Aviv University, Tel-Aviv, Israel; 6 Blueprint Genetics Oy, Helsinki, Finland; 7 The Meirav high risk clinic, Sheba Medical center, Tel Hashomer, Israel

**Keywords:** Cancer susceptibility genes, Double heterozygote, Mutant allele, Cancer phenotype, Age at cancer diagnosis

## Abstract

**Purpose:**

While cancer phenotypes in carriers of a single mutant allele in most major cancer susceptibility genes are well-established, there is a paucity of data on the phenotype of carriers of two pathogenic variants—double heterozygotes (DH) or homozygous carriers. Here, we describe the phenotype of carriers of homozygous and DH pathogenic sequence variants (PSVs) in major cancer susceptibility genes.

**Methods:**

Individuals referred for multigene panel testing at Blueprint Genetics laboratory were included. Ethically approved comparison of cancer type and age at diagnosis between DH, homozygous, and single PSV carriers was performed per gene.

**Results:**

Of 6,685 eligible participants, 928 (13.9%) were single heterozygous PSV carriers, 6 (0.09%) were homozygous PSV carriers, and 17 (0.25%) were DH PSV carriers. Mean age at diagnosis of any cancer among single PSV age was 46.8 ± 14.9 years and among DH PSV carriers 37.6 ± 13.0 years (*P* < 0.0001). Notably, age at diagnosis for breast cancer among single *BRCA1* PSV carriers (*n* = 59) was 43.8 ± 8.7 years (*p* = 0.7606), among single *BRCA2* PSV carriers (*n* = 52)—47.9 ± 13.0 years (*p* = 0.2274) compared with 42.3 ± 13.0 years among DH PSV carriers (*n* = 10- 9 of whom were carriers of either *BRCA1* or *BRCA2*).

**Conclusion:**

DH for PSV in two cancer susceptibility genes is a rare event, and the mean age at cancer diagnosis is younger in DH PSV carriers compared with single PSV carriers.

## Introduction

Individuals who carry a single heterozygous pathogenic sequence variant (PSV) in a major cancer susceptibility gene are at a significantly increased lifetime risk for developing cancer [1]. Cancer phenotype and age at cancer diagnosis vary by the mutated gene and other “modifier factors,” but a common theme to all high-risk cancer susceptibility genes is that the risk for developing cancer is several times higher than average risk in age stratified individuals [2]. Asymptomatic PSV carriers are counseled and provided with information regarding their cancer risk along with recommendations to mitigate that risk and facilitate early detection and/or active risk reduction, whenever possible (e.g., NCCN guidelines- https://www.nccn.org/guidelines/category_2). Cancer risk in asymptomatic PSV carriers and the suggested surveillance schemes together with risk-reducing measures are derived from a wealth of knowledge accumulated worldwide over the last 25 years and which have been implemented clinically as evident from their citations in clinical textbooks [3, 4]. These guidelines are published and adopted by national bodies such as NCCN (https://www.nccn.org/guidelines/category_2) NICE (https://www.nice.org.uk/about/what-we-do/our-programmes/nice-guidance/nice-guidelines) and other national and international organizations [e.g., ASCO (https://www.esmo.org/guidelines/guidelines-by-topic/hereditary-syndromes); ESMO (https://www.esmo.org/guidelines/guidelines-by-topic/hereditary-syndromes)]. While numerous studies encompass ethnically diverse populations that specify population-specific associated cancer risk in carriers of a single heterozygous PSV, there is a paucity of reported data on the malignant phenotype and specific cancer risks of double heterozygous (DH) PSV carriers (i.e., those who carry PSV in two different cancer susceptibility genes) and homozygous or compound heterozygote PSV carriers in the same gene [5]. This gap in research persists unless the condition is associated with a known recessive disease, such as Ataxia Telangiectasia or Fanconi anemia. Such information is vital to providing scientifically accurate oncogenetic counseling and consequent recommendations. In the current study, we evaluated the cancer phenotype of these rare cases detected using multi gene panel testing (MGPT) genotyped in a commercial laboratory.

## Methods

A cohort of patients referred for comprehensive cancer MGPT at Blueprint Genetics laboratory (Helsinki, Finland) were included. The criteria for referral varied and were determined by the referring physicians, including personal history of early diagnosed cancer, family history of cancer, or multiple cancer diagnoses in an individual and/or the family. These criteria were at the discretion of the referring physician and were not always specified in the submission forms.

Genetic analysis was carried out as previously described using next-generation sequencing technology [6] on DNA extracted using commercially available kits from buccal swabs (epithelial cells) or venous blood (leukocytes) and following the manufacturer’s protocol.

Anonymized data from all genotyped individuals were retrieved: age at genotyping, age range at cancer diagnosis (in 5-year strata), cancer type, the mutated genes, and specific PSV. This work was reviewed by the Western Institutional Review Board (IRB) and received an exemption determination.

Comparison between age at cancer diagnosis and cancer frequency and type in single PSV carriers, double PSV carriers, and homozygous PSV carriers were carried out using Pearson’s Chi-square test for categorical variables and linear model of two-way ANOVA with Dunnett’s correction for continuous variables using IBM SPSS Statistics for MAC, version 29.0 (IBM Corp., Armonk, N.Y., USA).

## Results

Overall, 6,685 individuals underwent genetic testing with a comprehensive hereditary cancer panel. Of these, 928 (13.9%) were single heterozygous PSV carriers, 17 (0.25%) were DH PSV carriers, and 6 (0.09%) were homozygous PSV carriers. The main clinical features of DH PSV carriers and the homozygous PSV carriers are shown in Table 1. Age at diagnosis and cancer phenotype in single heterozygous PSV carriers, DH PSV carriers, and homozygous PSV carriers were compared by gene (Table 2) and by cancer type (Table 3). Notably, in the DH PSV group, 16/17 (94.1%) had a personal history of cancer, as did all six patients in the homozygous PSV group, whereas in single PSV carrier group, only 30.4% (281/925) had a personal history of cancer. Mean age at diagnosis of any cancer was lower in the DH PSV group (37.6 ± 13.0 years, *p* = 0.023) and in the homozygous PSV group (17.2 ± 14.7 years, *p* < 0.001) compared with single PSV carrier group (46.8 ± 14.9 years). Notably, for patients with breast cancer, age at diagnosis in the DH PSV group (*n* = 10; 9 carriers of either *BRCA1* or *BRCA2*; 42.3 ± 13.0 years) was similar to the age at diagnosis among single *BRCA1* PSV carriers (*n* = 59, 43.8 ± 8.7 years, *p* = 0.76) and single *BRCA2* PSV carriers (*n* = 52, 47.9 ± 13.0 years, *p* = 0.17).

**Table 1:** Main clinical features of the patients with a homozygous pathogenic sequence variants (PSV) and patients with two PSVs

Zygosity	PSV in gene	Consequence of genetic change	Cancer type	Age group at dx (years)
Homozygous	MSH2	Nonsense	Lymphoma + glioblastoma	5–10
	MSH6	Nonsense	CRC + glioblastoma	20–25
	PMS2	Nonsense	ALL	5–10
	PMS2	Frameshift	Glioma, CRC, and 1 other	25–30
	PMS2	CNV deletion	Glioma	0–5
	CHEK2	Missense risk allele	BC	35–40
Heterozygous	BRCA2 CHEK2	Frameshift Frameshift	Gastric, BC	20–25
	ATM BRCA2	Frameshift Frameshift	BCx2, pancreatic, 3 others	25–30
	SDHA APC	Nonsense Frameshift	CRC	25–30
	PALB2 CHEK2	Frameshift CNV deletion	Papillary thyroid,	20–25
	BRCA2 DDX41	Frameshift Missense	BC, MDS	65–70
	ATM BRCA2	Frameshift Frameshift	BCx2	50–55
	ATM BRCA2	Frameshift Frameshift	BC	50–55
	CHEK2 ATM	Frameshift Alu insertion	Endometrial + lymphoma	45–50
	BRCA1 BARD1	Frameshift Frameshift	BC	50–55
	BRCA1 CHEK2	Frameshift Missense risk factor	BC	30–35
	BRCA2 MSH2	Frameshift Missense	CRC + paraganglioma	25–30
	MEN1 SDHA	Missense Splice donor	Paraganglioma	25–30
	BRCA2 CHEK2	Frameshift Splice donor	BC	35–40
	TP53 CHEK2	Missense Frameshift	BC	45–50
	BRCA2 CHEK2	Frameshift Frameshift	BC	35–40
	BRCA2 ATM FANCM	Frameshift Frameshift Nonsense	BC	25–30

*ALL* acute lymphatic leukemia, *BC* breast cancer, *BCx2* bilateral BC or local recurrence, *CRC* colorectal cancer, *MDS* myelodysplastic syndrome, *Dx* diagnosis

**Table 2:** Comparison of age at diagnosis between single heterozygous PSV carriers, DH PSV carriers and homozygous PSV carriers by gene

By gene/zygosity	gene	No. cases	Cancer type	Age group at dx-(years)	Mean age at dx- (years)	Mean at dx (years) (n)
Homozygous	MSH2	1	Lymphoma + glioblastoma	5–10		17.2 ± 14.7 (6)
	MSH6	1	CRC + glioblastoma	20–25		
	PMS2	3	ALL Glioma, CRCX2 Glioma	5–10 25–30 0–5		
	CHEK2	1	BC	35–40		
Double heterozygous	3 BRCA2 + ATM1 BRCA2 + ATM + FANCM	4	2 BC 1 BC × 2 1 BC × 2 + pancreatic ca + 3 others		40 ± 14.4	37.6 ± 13.0 (16)
	BRCA2 + CHEK2	3	2 BC 1 BC, gastric ca		32.5 ± 8.7	
	BRCA2 + MSH2	2	1 unaffected 1 CRC + paraganglioma	25–30		
	TP53 + CHEK2	1	BC	40–45		
	MEN1 + SDHA	1	Paraganglioma	30–35		
	BRCA1 + CHEK2	1	BC	30–35		
	BRCA1 + BARD1	1	BC	50–55		
	CHEK2 + ATM	1	Uterine + lymphoma	45–50		
	BRCA2 + DDX41	1	BC, MDS	65–70		
	PALB2 + CHEK2	1	Thyroid, BC	20–25		
	SDHA + APC	1	CRC	25–30		
Single heterozygous	BRCA1	72	Only affected individuals- all cancer phenotypes		43.4 ± 10.7	47.5 ± 14.9 (236)
	BRCA2	73			51.2 ± 14	
	MSH6	30			56.4 ± 12.1	
	ATM	27			48.6 ± 15.2	
	TP53	18			27.3 ± 19	
	MSH2	16			41.6 + 13.3

**Table 3:** Comparison of age at DX by cancer type between single DH and homozygous PSV carriers

Cancer type	BC (n/years)	CRC (n/years)	Gastric (n/year)	Pancreatic (n/years)	Uterine (n/years)
Homozygous	1 /35–40	2/25	–	–	–
Double heterozygous	10/42.3 ± 13.0	2/27.5	1/20–25	1 (4 different cancers, first dx BC at 25–30 years, pancreatic ca was the second diagnosed cancer	2/37.5
Single heterozygous	149/45.8 ± 11.9	25/46.9 ± 13.1	2/55	8/62.5 ± 10.7	23/59.5 ± 11.1

## Discussion

In the current study, the rates of detection of single, DH, and homozygous PSV carriers in cancer susceptibility genes were 13.9%, 0.25%, and 0.09%, respectively. The rates for DH should be compared with the few previously published studies that focused on these unusual cases. Infante et al. [7] reported DH rates of 1/623 when single genes were genotyped using Sanger sequencing, with a rate increasing to 3/116 (2.9%) when MGPT encompassing 35 genes was carried out. Njoroge et al. [8] report that in 0.2% of cases, an individual may be a DH for PSV in high-penetrance cancer gene. Our own unpublished data from the Meirav high-risk clinic show that 4/965 (0.4%) of *BRCA1* or *BRCA2* PSV carriers also have a PSV in *MSH2*, *MSH6*, or *TP53*. Weitzel and colleagues [9] reported that 47/55,803 (0.084%) cases genotyped for a 25-gene panel had a DH PSV in high-penetrance cancer genes. The reasons for the inconsistent rates across studies could be attributed to different selection criteria used for referral, the number of analyzed genes in each study, selection bias against women who were found to carry DH PSV in populations with founder PSVs, and the small number of analyzed individuals overall in most studies. Yet the common conclusion is that DH and homozygous PSV carriers are a rare event.

Indeed, most previously published studies reported a DH PSV in *BRCA1* and *BRCA2*, and hence assessing the effect of each gene on the resultant phenotype could not be clearly ascertained, given the overlap in phenotype in these two genes. Infante et al. [7] reported that age at diagnosis and severity of disease among *BRCA1/BRCA2* DH PSV carriers resembled *BRCA1* single PSV carrier features. Friedman et al. [10] reported that 5/ ~ 1500 cases (~ 0.3%) [all 5 cases representing 4 Ashkenazi Jewish (AJ) families] who were DH for one of the predominant AJ PSV in *BRCA1* and the predominant AJ PSV in *BRCA2*. The phenotype of these 5 patients was breast cancer (BC) at age 38 and 45 years, ovarian cancer at age 50 and 57 years, and one asymptomatic, cancer-free case at age 50 years. Vietri and coworkers [11] described 2 families with DH PSV in *BRCA1* and *BRCA2* and concluded that earlier age at BC diagnosis (32 years) and ovarian cancer diagnosis (36 years) imply that the phenotype is more severely affected by the *BRCA1* PSV. These authors reviewed the reported cases of *BRCA1/BRCA2* DH PSV worldwide up to 2020 {Table 6 in [11]}.

DH PSV in either the *BRCA1* or *BRCA2* genes with other cancer susceptibility genes or two non-*BRCA* cancer susceptibility genes have been reported less frequently, especially in populations not exhibiting founder PSVs. Infante et al. [7] reported one ovarian cancer case diagnosed at age 81 years who was a DH PSV carrier for *BRCA1/RAD51C,* and another case of BC (at age 40) and colorectal cancer (at age 54 years) who had DH PSV in *BRCA2/PMS2*. Weitzel and coworkers [9] reported that the most notable combinations of DH PSV in their large study were *BRCA1/PMS2, BRCA2/PMS2, BRCA1/BRCA2, BRCA1/MSH2, BRCA1/MSH6, BRCA2/MLH1,* and *BRCA2/MSH2* accounting for 28/40 (70%) DH PSV cases. Harada et al. [12] reported a Japanese family with Lynch Syndrome (LS) phenotype and ovarian cancer, where the proband was found to harbor PSV in both *MSH2* and *BRCA2*. Both PSVs were paternally inherited and were associated with LS phenotype rather than a *BRCA2* phenotype. Sorscher and coworkers [13] reported a single BC case diagnosed at age 52 years and a personal history of acute myeloid leukemia (AML) who carried DH PSV in *BRCA2/MLH1*. Tumor analysis revealed both allelic loss at the *BRCA2* locus and deficiency in mismatch repair gene protein expression (dMMR), indirectly implicating both genes as contributors to the tumorous phenotype in that case.

Ozer et al. [14] reported three cases who were DH PSV carriers in *BRCA* and one of the MMR genes: an AJ woman diagnosed with pancreatic cancer at age 57 harboring the predominant AJ germline PSV in both *BRCA1* and *MSH2* genes. The tumor did not display allelic loss at the *BRCA1* site, was microsatellite stable (MSS), and showed loss of protein expression of both MSH2 and MSH6 proteins. Another case was an Italian male diagnosed with pancreatic cancer at age 74 who harbored PSV in the *BRCA2* and *PMS2* genes. Notably, the family history was negative, and the tumor displayed allelic loss at the *BRCA2* locus and was MSS. A third case was a male presenting with metastatic rectal cancer at age 33, who harbored PSVs in *BRCA1* and in *MLH1*. The tumor was found to display high microsatellite instability (MSI-H), and MMR immunohistochemistry staining on rectal tumor biopsy showed absence of MLH1 and PMS2 protein expression. Tumor mutational burden was elevated, and allelic loss of the wild-type *MLH1* allele was found. Paternal history showed a father with colorectal cancer and the maternal history was consistent with the hereditary breast and ovarian cancer, though no formal oncogenetic testing was carried out in any parental family member.

Njoroge et al. [8] reported a case of a woman diagnosed with invasive lobular breast cancer at age 51 years, papillary thyroid cancer at age 52 years, and metastatic recurrence of breast cancer at age 65 with a family history of breast (maternal at age 74) and gastric (paternal at age 39) cancer. The proband was a DH PSV carrier in the *CDH1* and *PMS2* genes, which cause Hereditary Diffuse Gastric Cancer (and breast cancer) and Lynch syndrome, respectively. Laish et al. [15] reported that 11 individuals identified over a 15-year period in 3 oncogenetics services in Israel were DH PSV carriers of the AJ founder *BRCA* PSVs: *BRCA2* c.5946delT/c.6174delT (*n* = 10) or *BRCA1* c.68-69del/c.185delAG (*n* = 1). Four co-carried the *MSH2* c.1906G > C founder PSV, 3 co-carried the *MSH6* c.3984_3987dupGTCA, and 3 co-carried the *MSH6* c.3956_3957dup PSV. Eight double carriers (73%) had cancer: BC (5 cases, 2 bilateral), melanoma (2 cases), urothelial cancer (2 cases), and colon, endometrial, prostate, cutaneous squamous cell cancer, glioblastoma, gastric stromal tumor, and lymphoma (1 case each). Six carriers had 1–2 tumors, one had 3 tumors, and one had 5 primary tumors. The age at diagnosis of the first tumor ranged 36–76 years. These authors summarize all previously reported cases of BRCA-DNA MMR DH that were reported in the literature up to 2021 (*n* = 12) {Table 4 in [15]}. The recent report by van der Merwe et al. [16] stresses the fact that most of their DH PSV carrier cases (6/1600 – 0.375%) displayed TNBC and that the major phenotypic driver in their series are *BRCA1 BRCA2* PSVs.

Constitutional mismatch repair deficiency (CMMRD) is a rare childhood cancer predisposition syndrome (estimated at 1/1000000 live births), clinically hallmarked by high risk for developing high grade brain tumors and hematological malignancies, as well as some non-neoplastic features—such a cafe au lait spots [17]. The molecular basis for CMMRD is biallelic inactivating PVs in DNA MMR genes [18]. The specific cancer phenotype has been reported to be gene dependant: *MLH1*- and *MSH2*-associated CMMRD were associated with a higher hematological malignancies rate and earlier diagnosed, compared with *PMS2*-associated CMMRD where brain tumors were more frequent [18]. In the current study, the phenotypes of 5 genetically confirmed CMMRD cases are in line with the predicted phenotype, primarily since these were affected individuals with a clinical suspicion of CMMRD based on the type and age at cancer diagnoses,

In the current study, albeit limited in the number of DH and homozygous PSV carriers, it seems that age at any cancer diagnosis is significantly younger in DH PSV carriers compared with single PSV carriers.

The limitations of the current study should be enumerated. This is a single laboratory experience with a limited number of tested individuals including a minimal number of DH PSV carriers who were referred for testing for a variety of reasons. Another limitation of any conclusions that can be drawn from this study is the fact that the family data were not available for us and that the data on personal cancer status may have changed over the years.

In conclusion, a small proportion of individuals tested have DH for PSV in two cancer susceptibility genes, and the mean age at cancer diagnosis seems younger, compared with single PSV carriers.

## Data Availability

The datasets generated during and/or analyzed during the current study are not publicly available due to ethical restrictions, as per the ethics committee.
